# Williams–Campbell syndrome: an unusual presentation in an adult patient

**DOI:** 10.1259/bjrcr.20200052

**Published:** 2020-11-02

**Authors:** Mili Rohilla, Carlos Previgliano, Atefeh Geimadi, Guillermo Sangster

**Affiliations:** 1 Maulana Azad Medical College, New Delhi, India; 2 Department of Radiology, Louisiana State University Health Sciences Center, Shreveport, LA, USA

## Abstract

**Objective:**

Williams–Campbell syndrome (WCS) is a rare congenital disorder, which leads to bronchiectasis affecting fourth to sixth order of bronchial divisions. Symptoms include cough, sputum, wheeze and recurrent pulmonary infections, classically seen in the paediatric age group with selective bronchiectasis of the mid-order bronchioles. The literature describing diagnosis of Williams–Campbell syndrome in adult population is very sparse.

**Methods:**

This report presents a 62-year-old female with cough, fever, dyspnea and generalized body ache. She has had multiple admissions to the hospital since her childhood due to recurrent lower respiratory tract infections. Imaging findings demonstrated multiple cystic thin walled airways, compatible with bronchiectatic changes in the upper, middle and lower lobes bilaterally, bronchial wall thickening with air-fluid levels prominent in the fifth and sixth generation bronchial divisions, with normal calibre trachea and central bronchi. These radiological findings are consistent with diagnosis of Williams–Campbell syndrome, which was diagnosed after ruling out the other common causes of bronchiectasis.

**Conclusion:**

Williams–Campbell syndrome is a rare congenital cystic lung disease, the diagnosis of which is made by exclusion of common causes of bronchiectasis such as cystic fibrosis, allergic bronchopulmonary aspergillosis, tuberculosis, dyskinetic cilia syndrome and alpha-1 antitrypsin deficiency. Whenever the clinical picture is consistent with bronchiectasis, especially involving the mid-order bronchioles and recurrent pulmonary infections, it is wise to include WCS in the list of differential diagnoses, even in the adult population.

## Introduction

Williams–Campbell syndrome (WCS) is a rare congenital disorder due to deficiency of cartilage in subsegmental bronchi leading to the collapse of the distal airways and bronchiectasis or irreversible dilatation of bronchi and bronchioles, typically affecting the fourth to sixth order of bronchial divisions.^1,2^ Patients complain of bronchio-obstructive symptoms, such as cough, sputum, wheeze, and are prone to developing recurrent pulmonary infections. It classically presents in children with selective bronchiectasis of the mid-order bronchioles, sparing the central and most distal airways. Paracicatricial emphysema can be seen as emphysematous areas adjacent to areas of lung scarring. Patients usually have a normal-sized trachea and central bronchi.

The diagnosis is mainly clinical with classic radiological findings on chest CT with inspiratory and expiratory images and exclusion of other common entities causing bronchiectasis such as infections, cystic fibrosis, α-1 antitrypsin deficiency, hypogammaglobulinemia, allergic bronchopulmonary aspergillosis, tuberculosis, and sarcoidosis. Although a majority of the cases are reported amidst the paediatric age group, the literature describing the diagnosis of Williams–Campbell Syndrome is very sparse.^3–6^

## Clinical presentation

A 62-year-old female patient presented in the emergency department with complaints of acute onset shortness of breath, productive cough for the past two days, associated with fever, chills, generalized body aches and pleuritic chest pain. She is a lifetime non-smoker with no history of alcohol use. She has a past medical history of hypertension, gastro-esophageal reflux disease (GERD), benign paroxysmal positional vertigo (BPPV), asthma, osteoporosis and multiple hospital admissions due to recurrent pneumonia since her childhood, including diagnosed Nocardia and Moraxella infections and one episode of sputum positive for mycobacterium avium intracellulare in last 5 years. The patient moved to the city 5 years ago and did not have her prior medical records. She did not have any history of pulmonary tuberculosis (TB) or any known TB contacts. Her father had a history of emphysema.

On examination, her temperature was 100.5 °F/38.1°C, pulse rate was 112/min and blood pressure was 112/56 mm Hg. She was in respiratory distress with a respiratory rate of 18/min, SpO2 of 91% and expiratory wheezes. The examination of other systems was unremarkable.

## Investigations

Laboratory data showed an elevated white blood cell count of 13,500/µl with immature granulocytosis without peripheral blood eosinophilia. The basic metabolic panel revealed no abnormality. Respiratory viral infection panel by PCR was negative. The blood and urine sent for culture did not grow any organisms.

**Figure 1.**
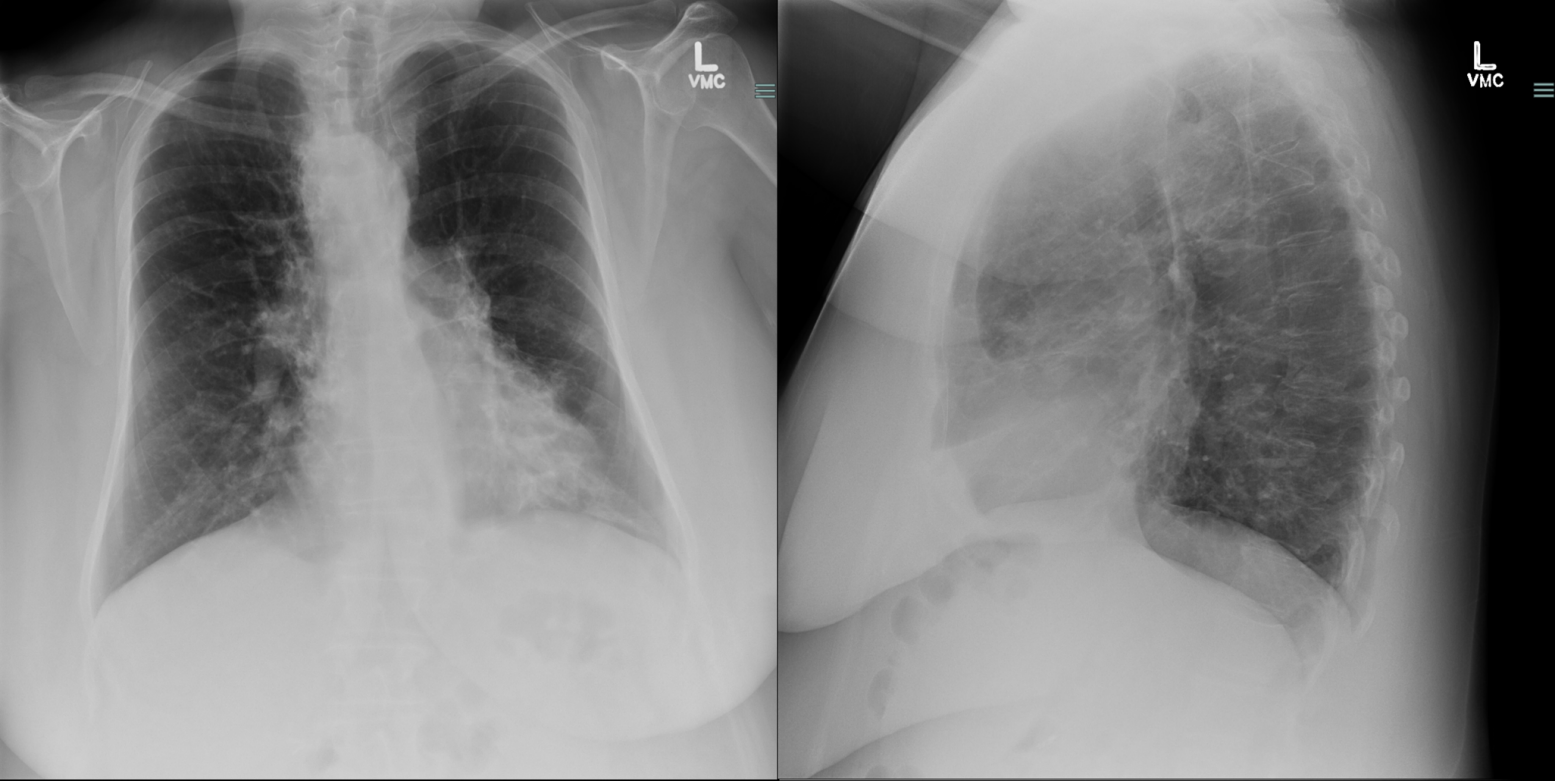
The posteroanterior and lateral chest radiographs demonstrated hyperexpansion of the lungs and bronchiectasis in the right suprahilar region and left lung base, along with presence of a right-sided aortic arch.

The posteroanterior and lateral chest radiographs demonstrated hyper-expansion of the lungs and bronchiectasis in the right suprahilar region and left lung base, along with the presence of a right-sided aortic arch. (Figure 1)

**Figure 2.**
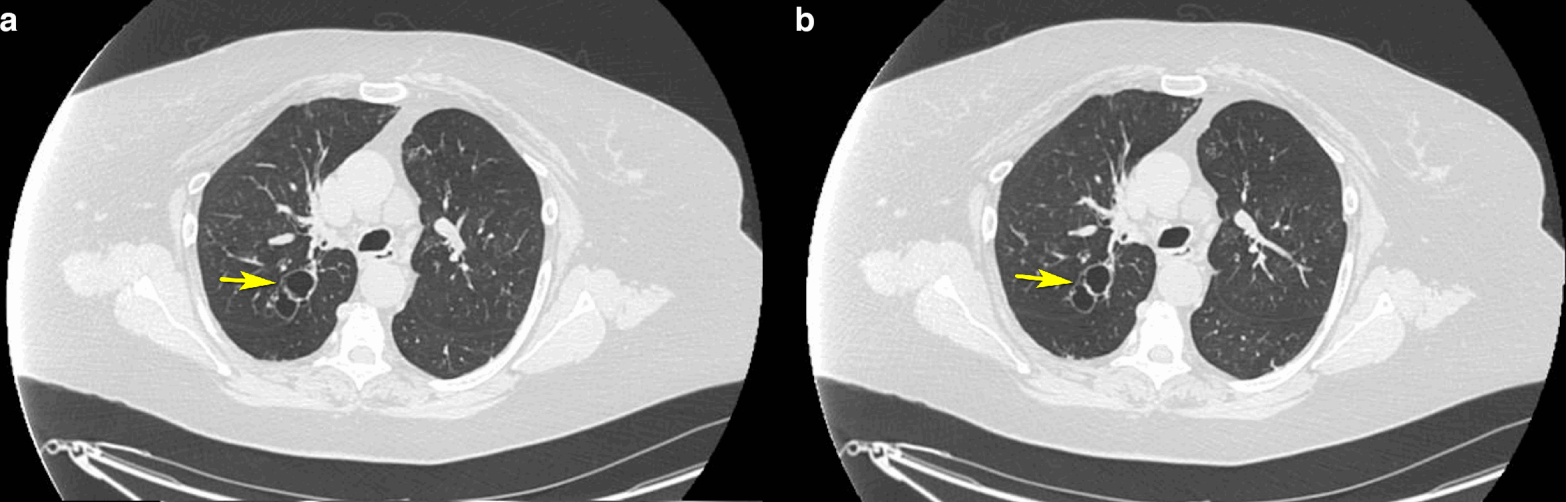
A and B. Axial non-contrast CT scan of the chest in inspiratory phase demonstrating hyperinflation of both lungs with multiple ballooning cystic bronchiectasis distal to third-generation bronchi bilaterally.

Axial non-contrast CT scan of the chest in the inspiratory phase demonstrated hyperinflation of both lungs with multiple ballooning cystic bronchiectasis distal to third-generation bronchi bilaterally. (Figure 2)

**Figure 3.**
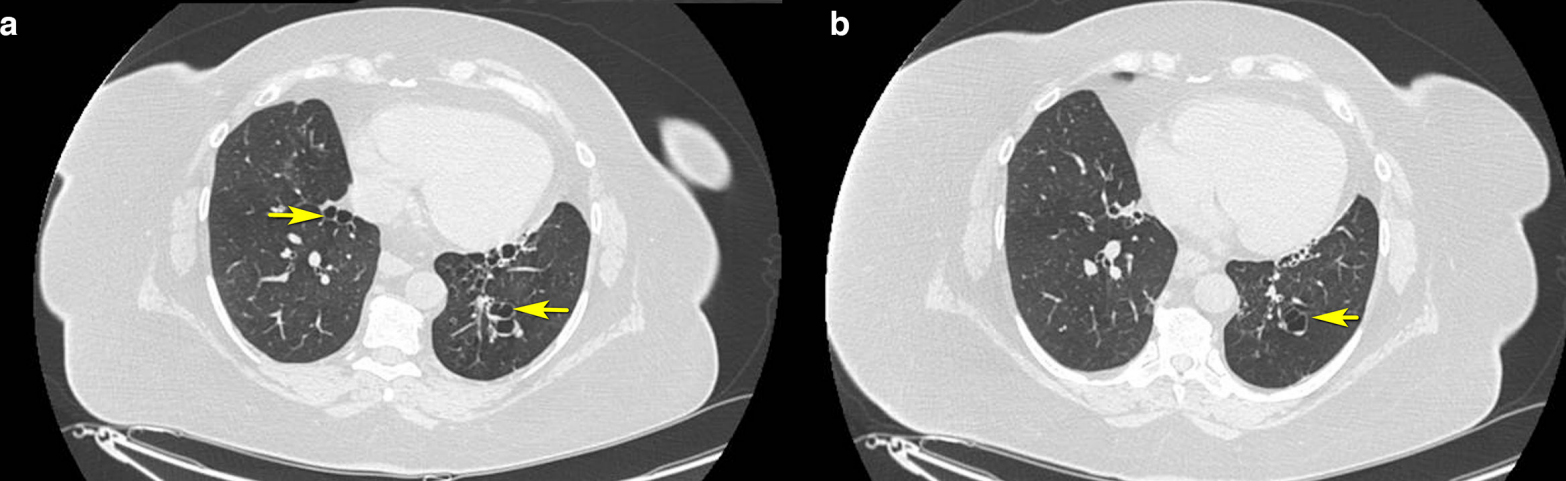
A and B. Axial non-contrast CT images of the chest in expiration showing the collapse of the bronchiectasis indicating the absence of cartilaginous plates in subsegmental bronchi.

The end-expiratory axial non-contrast CT scan of the chest showed the collapse of the bronchiectasis, indicating the absence of cartilaginous plates in subsegmental bronchi. (Figure 3)

**Figure 4.**
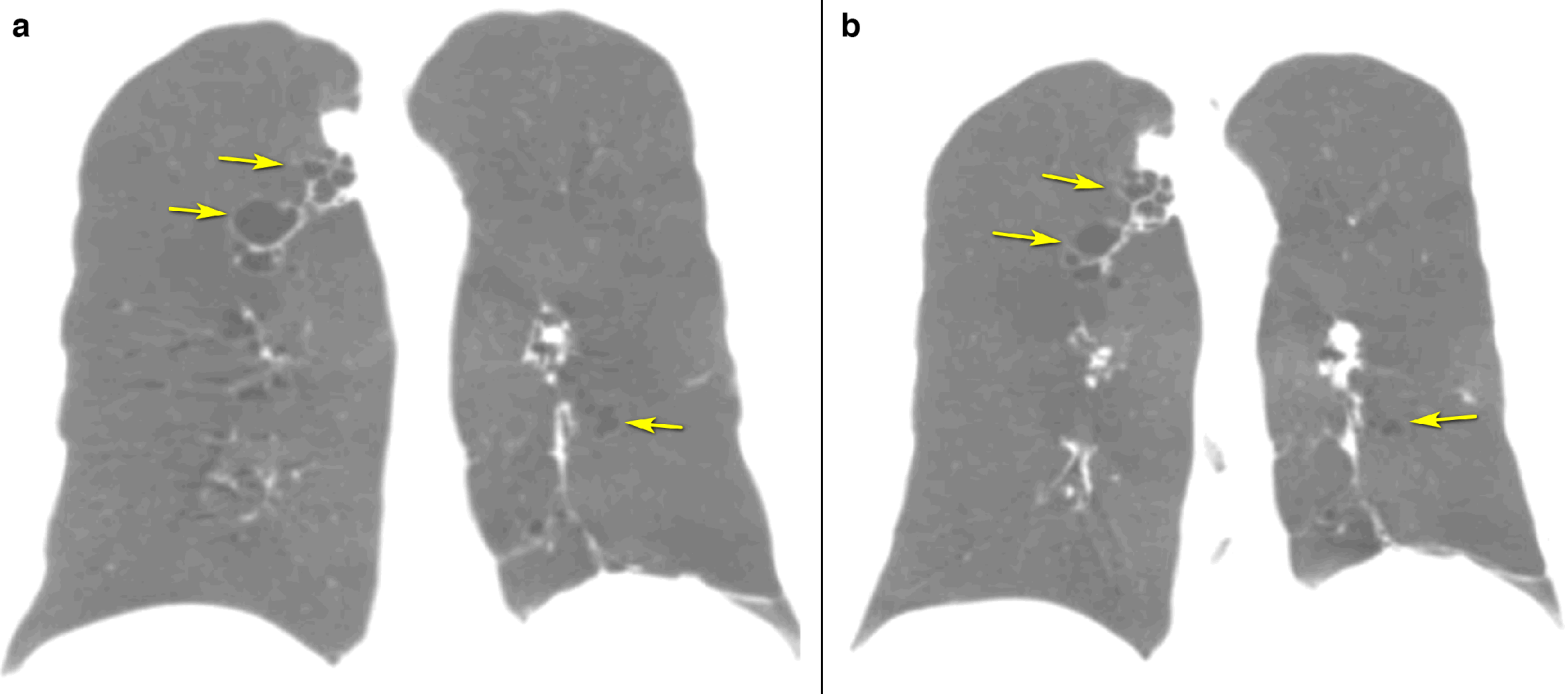
A. Coronal CT scan of the chest with minimal intensity projection (MinIP) in inspiration showing cystic bronchiectasis in fourth- to sixth-order bronchi in the right upper and left lower lobes with calcified hilar lymph nodes on the left- and right-sided aortic arch. B. Coronal CT scan of the chest with MinIP in the expiratory phase, demonstrating the collapse of the bronchi, a typical finding in this disease.

Coronal CT scan of the chest with minimal intensity projection (MinIP) in inspiration demonstrated cystic bronchiectasis in fourth- to sixth-order bronchi in the right upper and left lower lobes with calcified hilar lymph nodes on the left- and right-sided aortic arch, with no definite evidence of situs inversus. (Figure 4)

The collapse of the bronchi was noted on the expiratory Phase coronal chest CT with MinIP. (Figure 4)

**Figure 5.**
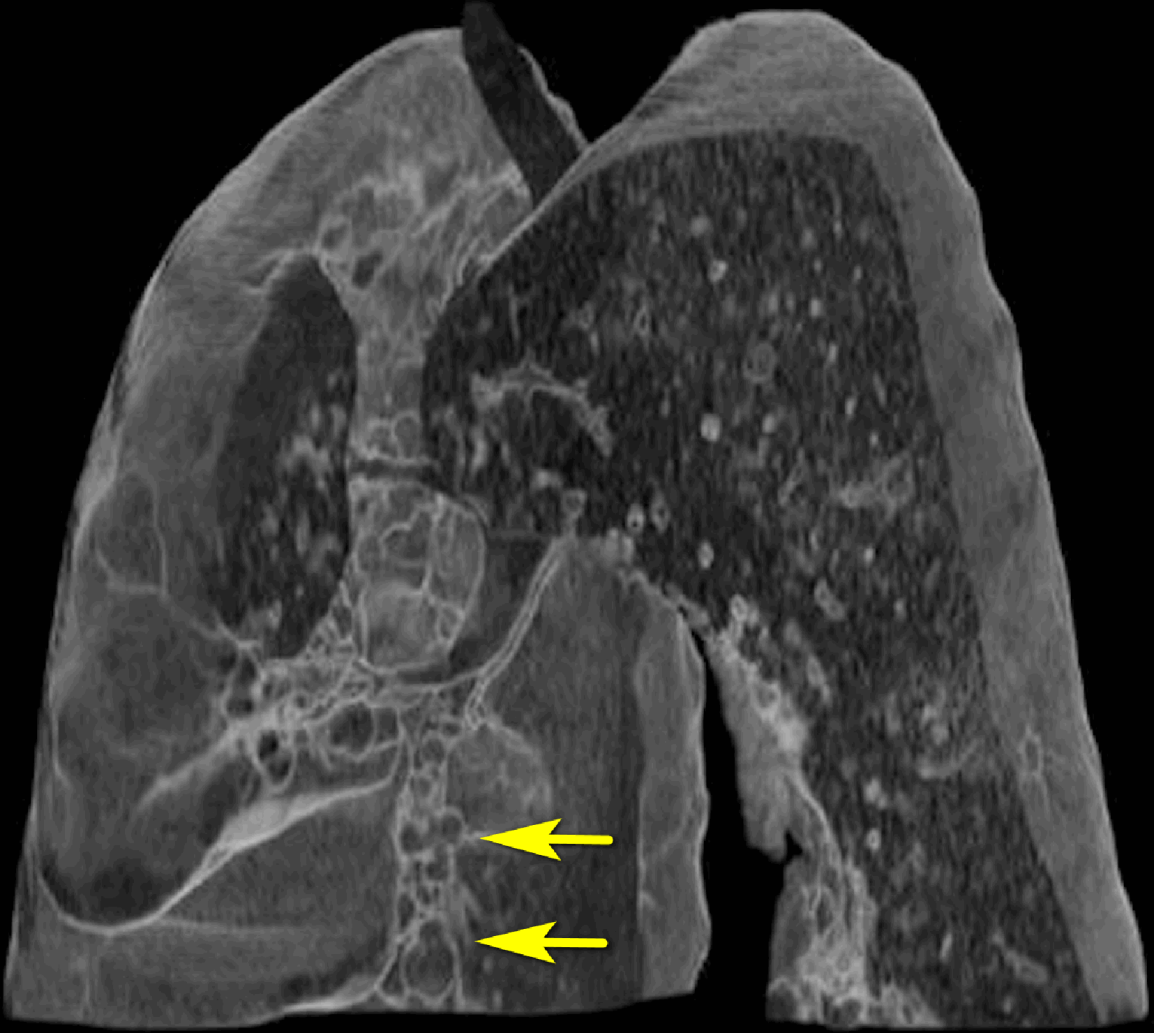
Oblique VR reconstruction shows cystic bronchiectasis in left lower lobe.

An oblique CT view of the chest was reconstructed using the VR software and demonstrated cystic bronchiectasis in the left lower lobe. (Figure 5)

Pulmonary Function Tests (PFTs) earlier demonstrated an obstructive pattern that has progressed to a fixed lung obstruction, as evidenced by a lack of reversibility on the post-bronchodilator spirometry.

Hypogammaglobulinemia was ruled out by quantification of serum IgG, IgA, IgE and IgM levels, which were normal. Serum α-1 antitrypsin levels were within the normal range. The sputum sample for acid-fast bacilli was negative. The sputum sample sent for fungal cultures did not report any growth. The sweat chloride test was negative. Anti-nuclear antibody and serum rheumatoid factors were negative. Anti Aspergillus immunoglobulins were within the normal range.

Based on the highly characteristic radiologic findings and the exclusion of other common causes of saccular bronchiectasis in the adult population, the patient was diagnosed with Williams–Campbell syndrome. She received treatment with chest physiotherapy, antibiotics, and acetylcysteine. Written informed consent was obtained from the patient for publication of the case report and accompanying images.

## Discussion

Williams–Campbell syndrome is an unusual form of bronchiectasis, described first in 1959 by Williams and Campbell in five children who presented with difficulty breathing, cough and recurrent respiratory infections.^2,7^ Chest imaging demonstrated thin-walled cystic bronchiectasis of the proximal bronchioles. A post-mortem study conducted on one of the children revealed that the fourth to the eighth generation of bronchioles had no cartilage, leading to Williams and Campbell’s postulation that the absence of cartilage in the bronchioles leads to bronchiectasis development and the subsequent symptoms in the paediatric population.

Multiple studies in the paediatric literature have been reported since then. However, a few studies did report Williams–Campbell syndrome in the adult population.^8,9^ The adult-variant of the disease can be attributed to patients with cartilage deficiency of a lesser degree, which may have been undiagnosed or presented late. Genetics and family history had been found to play a role in disease pathogenesis as well.^10^

The diagnosis is based on the classic presentation, symptoms, imaging findings and exclusion of other congenital and acquired causes of bronchiectasis (Table 1).^11^ The definitive diagnosis can only be made upon histopathological examination of the bronchial biopsy specimen demonstrating deficiency of bronchial cartilage. Since it is a high-risk invasive procedure, it is not recommended in antemortem patients for diagnosis. This finding can be correlated radiologically by demonstrating hyperinflation of lungs with bronchiectasis distal to third-generation bronchi bilaterally on the inspiratory film and the collapse of the bronchiectasis, indicating the absence of cartilaginous plates in subsegmental bronchi on the end-expiratory film.

**Table 1.** Common causes of bronchiectasis with their diagnostic evaluation methods

Aetiology of Bronchiectasis	Diagnostic Evaluation
***Congenital***	
**Tracheobronchial** (Bronchomalacia, Williams–Campbell Syndrome, Mounier–Kuhn Syndrome, Bronchial Cyst, Tracheo-Esophageal Fistula)	Chest CT with Inspiratory and Expiratory Images
**Vascular** (Pulmonary artery aneurysm, Pulmonary sequestration)	Chest CT Imaging
**Lymphatic** (Yellow-nail syndrome)	History of slow-growing, dystrophic nails
**Immunodeficiency Syndromes** (Bruton agammaglobulinemia, Common Variable Immunodeficiency, Selective IgA deficiency)	Quantitative immunoglobulin levels; immunoglobulin subclass levels
**Cystic fibrosis**	Sweat chloride; genetic testing
**Young’s syndrome** (Obstructive azoospermia with sinopulmonary infections)	Sperm Count
**Alpha-1 antitrypsin deficiency**	Alpha-1 antitrypsin serum level or genotyping
***Acquired***	
**COPD** (Chronic Bronchitis, Emphysema)	Pulmonary function tests
**Tumors** (Laryngeal papillomatosis; airway adenoma)	Chest imaging; flexible bronchoscopy
**Hilar adenopathy** (Tuberculosis; histoplasmosis; sarcoidosis)	PPD or IGRA; Chest HRCT
**Mucoid impaction** (Allergic bronchopulmonary aspergillosis, postoperative mucoid impaction)	Total serum IgE, Aspergillus specific IgE and IgG; Aspergillus skin test; Chest imaging
**Recurrent aspiration pneumonia** (Alcoholism; neurologic disorders)	History; Chest imaging
**Rheumatic disease** (Rheumatoid Arthritis, Sjogren syndrome, IBD)	History; Rheumatoid factor; Antinuclear antibody, antiSSA/ antiSSB
**Infections** (Bacterial, Viral, Fungal)	History of infection; sputum culture; Serology; Fungal culture; AFB smear; Mycobacterial Culture

CT: Computed Tomography; Ig: Immunoglobulin; COPD: Chronic Obstructive Airway Disease; PPD: Purified Protein Derivative; IGRA: Interferon γ release assay.

Another differential to be considered can be Mounier–Kuhn syndrome, a rare entity characterized by dilated trachea, bronchi and recurrent lower respiratory tract infections, due to atrophy of muscles and elastic tissue in the trachea and bronchi. It can be diagnosed when the diameter of the trachea (measured 2 cm above the main carina), right mainstem bronchus and left mainstem bronchus exceeds a diameter of two standard deviations above the normal.^12^

The characteristic radiological findings of WCS include lung hyperinflation and cystic bronchiectasis classically in the fourth to the sixth generation of bronchioles with normal trachea and central bronchi.^13^

The clinical course can be variable, depending on the degree of cartilage involvement, exposure to infectious organisms, genetic factors, and individual immune response. It can vary from severe disease and early death in some children to prolonged survival in some adult patients.^14^ The prognosis in the adult population still remains unknown.

There is no specific treatment for Williams–Campbell syndrome, and prophylaxis using antibiotics remains the mainstay of the treatment.^15^ Symptomatic treatment includes chest physiotherapy to aid in the mobilization of secretions, bronchodilators, oxygen therapy, positive pressure ventilation and drainage if necessary.^16^

Bronchiectasis is a leading cause of morbidity among adults in the USA, which is significantly more common in females as compared to males, with an estimated 70,000 adults newly diagnosed with bronchiectasis each year in the USA.^17^ Since bronchiectasis is an important public health issue, requiring utilization of extensive healthcare resources such as frequent ambulatory visits, antibiotic usage, chest CT and hospitalization, it is imperative to diagnose the correct aetiology and provide focused management for the patients.^18^

In the aforementioned case, the features suggestive of WCS include recurrent pulmonary infections since childhood, no known history of pulmonary tuberculosis, smoking or alcohol intake, and chest CT findings demonstrating hyperinflation of both lungs with cystic bronchiectasis distal to third-generation bronchi bilaterally in the end-inspiratory films and collapse of the bronchiectasis indicating the absence of cartilaginous plates in subsegmental bronchi in the end-expiratory films. The peripheral blood picture with neutrophil-predominant leukocytosis corroborates the bronchiectatic aetiology as airway neutrophils, and the unopposed activity of neutrophil-elastase contributes to the disease pathogenesis.^19^ The other differentials of bronchiectasis, mentioned in Table 1, were ruled out using chest CT scan, pulmonary function tests, PCR for respiratory viral infections, quantification of serum immunoglobulins, sweat chloride test, serum alpha-1 antitrypsin levels, sputum mycobacterial and fungal cultures, rheumatoid factor, anti nuclear antibody, and anti aspergillus immunoglobulins. The authors believe that the late presentation of the patient at the age of 62 years can be attributed to the milder degree of cartilaginous deficiency leading to slower symptom progression in addition to decreased utilization of healthcare facilities and lack of available patient data before she moved to the city 5 years ago.

## Learning points

- Williams–Campbell syndrome is an uncommon cause of recurrent pulmonary infections in the adult population, characterized by cystic, saccular bronchiectasis in mid-order bronchioles due to deficiency of bronchial cartilage.
- Whenever the clinical and radiologic findings in an adult suggest productive cough, dyspnea, recurrent lower respiratory tract infections, and bronchiectasis, Williams–Campbell syndrome should be included in the list of differential diagnoses.
- The common causes of cystic bronchiectasis include cystic fibrosis, tuberculosis, allergic bronchopulmonary aspergillosis, hypogammaglobulinemia and immotile cilia syndrome.
- A chest CT scan with inspiratory and expiratory films provides helpful information in making the diagnosis.
